# Gender, marital and educational inequalities in mid- to late-life depressive symptoms: cross-cohort variation and moderation by urbanicity degree

**DOI:** 10.1136/jech-2020-214241

**Published:** 2020-11-04

**Authors:** Milagros A Ruiz, Marielle A Beenackers, Dany Doiron, Asli Gurer, Aliou Sarr, Nazmul Sohel, Erik J Timmermans, Rita Wissa, Basile Chaix, Martijn Huisman, Steinar Krokstad, Ruzena Kubinova, Sofia Malyutina, Parminder Raina, Abdonas Tamosiunas, Frank J van Lenthe, Martin Bobak

**Affiliations:** 1 Research Department of Epidemiology and Public Health, University College London, London, UK; 2 Department of Public Health, Erasmus University Medical Center, Rotterdam, Netherlands; 3 Research Institute of the McGill University Health Centre, Montreal, Canada; 4 Department of Health Research Methods, Evidence and Impact, McMaster University, Hamilton, Ontario, Canada; 5 McMaster Institute for Research on Aging, Hamilton, Ontario, Canada; 6 Department of Epidemiology and Biostatistics, Amsterdam UMC, VU University Medical Center, Amsterdam Public Health Research Institute, Amsterdam, Netherlands; 7 Sorbonne Université, INSERM, Institut Pierre Louis d'Epidémiologie et de Santé Publique, Nemesis Research Team, Paris, France; 8 Department of Sociology, Faculty of Social Sciences, Vrije Universiteit Amsterdam, Amsterdam, Netherlands; 9 HUNT Research Centre, Department of Public Health and General Practice, Faculty of Medicine, Norwegian University of Science and Technology (NTNU), Levanger, Norway; 10 Levanger Hospital, Nord-Trøndelag Hospital Trust, Levanger, Norway; 11 Centre for Environmental Health Monitoring, National Institute of Public Health, Prague, Czech Republic; 12 Research Institute of Internal and Preventive Medicine, Branch of the Institute of Cytology and Genetics, SB RAS, Novosibirsk, Russia; 13 Novosibirsk State Medical University, Novosibirsk, Russia; 14 Institute of Cardiology, Academy of Medicine, Lithuanian University of Health Sciences, Kaunas, Lithuania; 15 Faculty of Public Health, Academy of Medicine, Lithuanian University of Health Sciences, Kaunas, Lithuania; 16 Department of Human Geography and Spatial Planning, Utrecht University, Utrecht, Netherlands

**Keywords:** Ageing, urbanisation, social epidemiology, depression

## Abstract

**Background:**

Although ageing populations are increasingly residing in cities, it is unknown whether depression inequalities are moderated by urbanicity degree. We estimated gender, marital and educational inequalities in depressive symptoms among older European and Canadian adults, and examined whether higher levels of urbanicity, captured by population density, heightened these inequalities.

**Methods:**

Harmonised cross-sectional data on 97 826 adults aged ≥50 years from eight cohorts were used. Prevalence ratios (PRs) were calculated for probable depression, depressed affect and depressive symptom severity by gender, marital status and education within each cohort, and combined using random-effects meta-analysis. Using a subsample of 73 123 adults from six cohorts with available data on population density, we tested moderating effects measured by the number of residents per square kilometre.

**Results:**

The pooled PRs for probable depression by female gender, unmarried or non-cohabitating status and low education were 1.48 (95% CI 1.28 to 1.72), 1.44 (95% CI 1.29 to 1.61) and 1.29 (95% CI 1.18 to 1.41), respectively. PRs for depressed affect and high symptom severity were broadly similar. Except for one Dutch cohort with findings in an unexpected direction, there was no evidence that population density modified depressive symptom inequalities.

**Conclusions:**

Despite cross-cohort variation in gender, marital status and educational inequalities in depressive symptoms, there was weak evidence that these inequalities differed by levels of population density.

## INTRODUCTION

Globally, years lived with disability owing to depressive disorders increased by 38% from 1990 to 2010.^1 2^ Major depressive disorder (MDD) occurs in 1–5% of adults aged ≥65 years worldwide; and clinically significant depressive symptoms, which fall below the diagnostic criteria for MDD, afflict 15% of adults aged ≥65 years.^2^ Being female, unmarried/divorced/widowed, and having low education are potent risk factors for depressive disorders in later life.^3–5^ Prior studies, however, have shown that these depression inequalities vary between countries,^6–8^ which may be attributable to differences in social environments.

Parallel to the rise of mid- to late-life depression, the share of the global population living in cities rose from 43% to 54% from 1990 to 2014, and will reach 66% by 2050. As the pace of urbanisation has accelerated, mental disorders have increased among city dwellers.^9^ Recent findings indicate that depression risk is 44% higher in older urban adults than in rural counterparts in developed countries.^2^ The impact of these urban–rural differences are considerable, since 43% of older adults from developed societies currently reside in cities.^10^

Gender, marital status and education, on the one hand,^3–5^ and urban living,^2^ on the other hand, have been extensively studied in relation to mid- to late-life depression. Despite the growing importance of the urbanised world for ageing populations, to our knowledge, no study has examined whether urbanicity, defined as the presence of conditions specific to urban areas,^9^ interacts with older adults’ position in society to influence their mental health. First, older people’s safety in cities, where injuries and crime are rampant,^9^ is associated with psychological distress, but effects appear twice as high in older women compared to older men.^11^ Second, social relationships, which protect against mid- to late-life depression, appear weaker at higher levels of population density. Social cohesion and collective efficacy seem weaker in more dense communities, and older adults living in these areas tend to have more numerous, but less intimate, personal relationships.^12^ The social integration of older singletons and widowers may be poorer in more urbanised settings.^8^ Third, although dense cities often have better service environments than smaller cities and non-urban areas,^9^ health and social services can often be overburdened, which could unequally affect socially disadvantaged adults.

Since mid- to late-life depression inequalities in different settings may vary by urbanicity degree, we measured cross-sectional associations of gender, marital status and education with depressive symptoms among older adults in Europe and Canada, and tested whether population density, a marker of urbanicity,^9^ modified the hypothesised inequalities. As urbanicity refers to the conditions which are far more predominant in urban areas than non-rural areas,^9^ population density is often used to examine the impact of urban living on health.^13^

## METHODS

### Study populations

Harmonised data from the Promoting Mental Well-being in the Ageing Population: Determinants, Policies and Interventions in European Cities (MINDMAP) project from eight cohort studies^14^ were analysed (in descending analytic sample size): the Canadian Longitudinal Study on Aging^15^ (CLSA, n=45 782); the Nord-Trøndelag Health Study^16^ (HUNT, n=23 571); the Russian (HAPIEE-RU, n=7766), Czech (HAPIEE-CZ, n=6726) and Lithuanian (HAPIEE-LT, n=6239) cohorts from the Health, Alcohol and Psychosocial factors In Eastern Europe multi-country study^17^; the Residential Environment and CORonary heart Disease study^18^ (RECORD, n=3706); and the Longitudinal Aging Study Amsterdam^19^ first (LASA-1, n=3041) and second (LASA-2, n=995) cohorts.

The analytic sample (n=97 826) comprised adults aged ≥50 years with complete data on depressive symptoms, gender, marital status and education plus covariates at Wave 1 for all cohorts except HUNT (Wave 2). An analytic subsample (n=73 123) also comprised adults aged ≥50 years with the aforementioned data plus information on population density from six studies at Wave 1 (CLSA=45 782; RECORD=3706), Wave 2 (HAPIEE-CZ=1241; LASA-2=835), Wave 3 (HUNT=20 566) or Wave 5 (LASA-1=993). Selection criteria of both samples are depicted in flow diagrams (online supplemental figure S1).

10.1136/jech-2020-214241.supp1 Supplementary data

### Depressive symptoms

Four depression screening scales designed for general populations, including older adults, were administered across cohorts: the Center for Epidemiological Studies Depression (CES-D) scale—10-item version (CLSA-COP,^20^ CLSA-TRA^20^ and HAPIEE-LT^21^) and the CES-D 20-item version^22^ (HAPIEE-RU, HAPIEE-CZ, LASA-1 and LASA-2), the 7-item depression sub-scale from the Hospital Anxiety and Depression Scale^23^ (HADS-D) (HUNT) and the 13-item Questionnaire of Self-Evaluated Depressive Symptomatology^24^ (QD2A) (RECORD). Depressive symptom scores were used to derive study-specific measures of probable depression and depressive symptom severity. Probable depression was based on whether participants had elevated depressive symptoms at or above the threshold for the specific CES-D 10,^20 21^ CES-D 20,^25^ HADS-D^23 26^ and QD2A^24^ scores. Since higher scores are intended to reflect higher depression risk, scores were split into study-specific tertiles denoting low, moderate or high severity. Depressed affect was based on whether participants reported feeling sad, depressed or downhearted. Online supplemental table S1 reports the retrospective data harmonisation performed to derive these outcomes.

### Covariates

Covariates included age, age squared, gender, marital or cohabitation status, and education classified as high (postsecondary non-tertiary or higher, ISCED 4–8) or low (upper secondary or lower, ISCED 0–3), using the International Standard Classification of Education (ISCED 2011).^27^ Self-rated health was also included as a confounder to control for potential country differences in reporting behaviour.^28^ Population density of participants’ residence, defined as the number of residents per square kilometre, was matched the studies’ examination period as closely as feasibly possible: 2016 data were linked to CLSA Wave 1 (2010–2015), 2011 data to HAPIEE-CZ Wave 2 (2006–2008), 2009 data to RECORD Wave 1 (2007–2008) and 2006 data to HUNT Wave 3 (2006–2008) as well as LASA-1 Wave 5 and LASA-2 Wave 2 which were concurrently examined in 2005–2006. Population density data were provided by Statistics Canada (CLSA), Statistics Netherlands (LASA-1 and LASA-2), Statistics Norway (HUNT), the Czech Statistical Office (HAPIEE-CZ) and the National Institute of Statistics and Economic Studies (RECORD), which were calculated at the census subdivision level (CLSA), municipality level (HUNT and RECORD) and neighbourhood level (in the remaining cohorts).

### Statistical analyses

Prevalence proportions of depressive symptoms were directly age-standardised using the WHO 2013 European Standard Population, and estimated by gender, marital status and education in each cohort. Generalised linear models with a binomial family distribution and log link function calculated prevalence ratios (PRs) for probable depression and depressed affect. Multinomial logistic regression calculated odds ratios for moderate and high versus low symptom severity, which were converted into PRs.^29^ For each outcome, cohort-specific PRs were calculated using a one-step approach that adjusted for gender, marital status, education, plus age, age squared and self-rated health. Cohort-specific PRs by gender, marital status and education were combined to calculate pooled PRs by each determinant using random-effects meta-analysis. The I^2^ statistic described the proportion of between-cohort heterogeneity in associations that are not attributable to sampling variation.

In addition, we tested whether population density modified the associations described earlier in the subsample of six cohorts using two-way interaction terms between each determinant and population density. Effects were assessed for every 1000 increase in residents per square kilometre in all cohorts except for HUNT, where effects were quantified for every 10 residents per square kilometre since the maximum population density was 32 residents per square kilometre.

Cohort-specific analyses and meta-analyses were conducted using R Studio and Stata 15, respectively. CLSA analyses incorporated sampling weights to reflect the Canadian population aged 45–85 years.^15^

## RESULTS

Women comprised over half of the cohort samples, except in RECORD (36.2%) (table 1). The share of unmarried/non-cohabitating adults ranged from 16.2% (HUNT) to 36.3% (LASA-1). Adults having low education ranged from 29% in CLSA to 88% in LASA-1, reflecting generational differences in formal education as these cohorts were born in 1960 or earlier, and 1942 or earlier, respectively. Age-standardised prevalence of probable depression and depressed affect in the Lithuanian and Russian HAPIEE cohorts ranged from 29.1% to 32.3% and from 18.8% to 34.1%, respectively, compared to <5% in HUNT. Urbanicity was highest in RECORD as half of the sample lived in areas with 10 829 residents per square kilometre. Median population density ranged from 4700 to 5200 in the LASA cohorts, and fell to 2000 and 1000 km^2^ residents in HAPIEE-CZ and CLSA, respectively. Population density was remarkably low in HUNT with a median of 13 residents per square kilometre.

**Table 1:** Study-specific characteristics of analytic cohort samples

Main analytic sample (n=97 826)	Country and cohort							
	CA	NO	RU	CZ	LT	FR	NL	NL
	CLSA	HUNT	HAPIEE	HAPIEE	HAPIEE	RECORD	LASA-1	LASA-2
Number of participants	45 782	23 571	7766	6726	6239	3706	3041	995
Data collection wave (year)	Wave 1 (2010–2015)	Wave 2 (1995–1997)	Wave 1 (2002–2005)	Wave 1 (2002–2005)	Wave 1 (2006–2008)	Wave 1 (2007–2008)	Wave 1 (1992–1993)	Wave 1 (2002–2003)
Mean years of age (SD)	62.4 (9.3)	64.3 (10.0)	59.8 (5.8)	59.7 (5.8)	62.2 (6.3)	59.7 (7.1)	70.2 (8.8)	59.4 (3.0)
Female, %	51.5	52.9	53.8	52.7	54.5	36.2	51.6	52.6
Unmarried/non-cohabitating, %	25.9	15.9	29.1	24.4	31.2	33.4	36.3	20.3
Low education, %	29.1	72.3	71.5	86.1	45.4	54.3	88.5	78.3
Fair/poor self-rated health, %	11.8	26.6	89.7	61.2	76.0	46.0	37.7	33.1
Age-standardised prevalence of probable depression, %	16.7	4.5	29.1	19.2	32.3	7.5	11.8	7.4
Age-standardised prevalence of depressed affect, %	8.5	2.4	18.8	8.2	34.1	14.1	5.0	2.1
Age-standardised prevalence of depressive symptom severity, %								
Low	31.2	23.1	41.3	31.4	43.0	47.6	38.8	28.1
Moderate	37.2	37.2	20.5	31.6	24.7	30.1	36.6	50.6
High	31.6	39.7	38.2	37.0	32.3	22.3	24.6	21.3
**Analytic subsample (n=73 123)**								
Number of participants	45 782	20 566	–	1241	–	3706	993	835
Data collection wave (year)	Wave 1 (2010–2015)	Wave 3 (2006–2008)	–	Wave 2 (2006–2008)	–	Wave 1 (2007–2008)	Wave 5 (2005–2006)	Wave 2 (2005–2006)
Number of residents per square kilometre								
Mean (SD)	1056.8 (1358.8)	14.1 (8.7)	–	2030.0 (1383.1)	–	14 689.9 (11 332.6)	4723.9 (3972.5)	5185.3 (4845.1)
25th percentile	82	8	–	1030	–	5379	2686	2786
50th percentile	476	13	–	1435	–	10 829	4228	4494
75th percentile	1484	21	–	3251	–	25 459	6018	6126

CA, Canada; CLSA, Canadian Longitudinal Study on Aging; CZ, The Czech Republic; FR, France; HAPIEE, the Health, Alcohol and Psychosocial factors In Eastern Europe; HUNT, the Nord-Trøndelag Health Study; LASA-1, Longitudinal Aging Study Amsterdam—first cohort; LASA-2, Longitudinal Aging Study Amsterdam—second cohort; LT, Lithuania; NL, The Netherlands; NO, Norway; RECORD, the Residential Environment and CORonary heart Disease study, RU, Russia.

### Differences in age-standardised prevalence of depressive symptoms

Women, unmarried/non-cohabitating adults and adults with low educational attainment reported a greater prevalence of probable depression, depressed affect (table 2) and depressive symptom severity (online supplemental table S2), although the differences in prevalence by gender, marital status and education varied between cohorts.

**Table 2:** Age-standardised study-specific prevalence of probable depression and depressed affect by gender, marital status and education

Country and cohort		Male	Female	Married/cohabitating	Not married/cohabitating	High education	Low education
**Probable depression** **(%)**							
CA	CLSA	13.51 (12.76 to 14.27)	19.50 (18.65 to 20.36)	14.06 (13.37 to 14.75)	23.65 (22.52 to 24.78)	15.44 (14.77 to 16.11)	19.39 (18.26 to 20.51)
NO	HUNT	4.35 (3.94 to 4.76)	4.63 (4.23 to 5.03)	4.25 (3.94 to 4.56)	5.22 (4.49 to 5.95)	2.79 (2.29 to 3.28)	4.98 (4.64 to 5.33)
RU	HAPIEE	19.17 (16.91 to 21.42)	37.72 (35.20 to 40.23)	24.39 (22.39 to 26.38)	40.14 (36.91 to 43.37)	23.61 (20.48, 26.73)	31.10 (29.07, 33.14)
CZ	HAPIEE	13.85 (12.29 to 15.41)	24.69 (22.40 to 26.99)	16.30 (14.89 to 17.70)	27.67 (24.70 to 30.64)	13.82 (10.76 to 16.87)	20.11 (18.65, 21.58)
LT	HAPIEE	25.40 (24.18 to 26.63)	29.76 (28.19 to 31.33)	18.39 (17.19 to 19.58)	42.07 (40.10 to 44.04)	20.76 (19.36 to 22.16)	34.91 (33.39 to 36.42)
FR	RECORD	5.16 (4.12 to 6.20)	10.90 (9.10 to 12.70)	6.52 (5.45 to 7.60)	9.56 (7.51 to 11.61)	5.44 (4.13 to 6.74)	9.05 (7.66 to 10.44)
NL	LASA-1	8.95 (7.39 to 10.51)	14.33 (12.63 to 16.02)	8.66 (7.00 to 10.33)	23.27 (20.19 to 26.36)	10.48 (7.17 to 13.79)	11.89 (10.65 to 13.13)
NL	LASA-2	5.75 (4.09 to 7.42)	9.97 (8.26 to 11.68)	6.02 (4.88 to 7.15)	25.32 (19.32 to 31.33)	5.66 (3.21 to 8.11)	8.20 (6.90 to 9.51)
**Depressed affect** **(%)**							
CA	CLSA	7.01 (6.47 to 7.56)	9.79 (9.13 to 10.46)	7.33 (6.83 to 7.82)	11.62 (10.76 to 12.49)	7.88 (7.37 to 8.39)	9.90 (9.04 to 10.76)
NO	HUNT	1.99 (1.73 to 2.25)	2.82 (2.51 to 3.12)	2.29 (2.06 to 2.53)	3.48 (2.84 to 4.11)	1.73 (1.39 to 2.07)	2.70 (2.45 to 2.96)
RU	HAPIEE	12.39 (10.34 to 14.44)	24.41 (22.08 to 26.74)	15.55 (13.78 to 17.33)	26.69 (23.62 to 29.75)	13.25 (10.72, 15.77)	20.88 (18.99 to 22.77)
CZ	HAPIEE	5.60 (4.49 to 6.71)	10.63 (9.19 to 12.08)	6.60 (5.67 to 7.53)	13.10 (10.94 to 15.25)	4.73 (3.46 to 6.00)	8.84 (7.81 to 9.88)
LT	HAPIEE	27.04 (25.76 to 28.32)	31.87 (30.27 to 33.48)	20.61 (19.36 to 21.85)	43.15 (41.17 to 45.14)	22.81 (21.36 to 24.27)	36.43 (34.90 to 37.97)
FR	RECORD	10.05 (8.72 to 11.39)	20.14 (17.86 to 22.41)	13.38 (11.93 to 14.84)	15.72 (13.31 to 18.13)	11.25 (9.53 to 12.97)	16.26 (14.51, 18.02)
NL	LASA-1	2.65 (1.91 to 3.40)	7.07 (5.78 to 8.36)	2.88 (2.23 to 3.53)	11.31 (8.95 to 13.67)	2.39 (0.93 to 3.84)	5.31 (4.44 to 6.18)
NL	LASA-2	1.38 (0.53 to 2.23)	2.98 (1.98 to 3.99)	1.51 (0.91 to 2.11)	8.60 (4.78 to 12.41)	0.60 (−0.23 to 1.43)	2.53 (1.77 to 3.30)

CA, Canada; CLSA, Canadian Longitudinal Study on Aging; CZ, The Czech Republic; FR, France; HAPIEE, the Health, Alcohol and Psychosocial factors In Eastern Europe; HUNT, the Nord-Trøndelag Health Study; LASA-1, Longitudinal Aging Study Amsterdam—first cohort; LASA-2, Longitudinal Aging Study Amsterdam—second cohort; LT, Lithuania; NL, The Netherlands; NO, Norway; PR, prevalence ratio; RECORD, the Residential Environment and CORonary heart Disease study, RU, Russia.

The exception was HUNT, where high symptom severity was slightly more prevalent in men than in women. Prevalence differences among women relative to men were particularly high in HAPIEE-RU: 19% for probable depression and high symptom severity and 12% for depressed affect.

Prevalence differences between marital/cohabitating groups were again smallest in HUNT, and greatest at over 20% in HAPIEE-LT for probable depression and depressed affect. For high symptom severity, the differentials were high in the LASA cohorts (24–30%) and HAPIEE-LT (24%).

Educational inequalities were seen in all cohorts. For probable depression, prevalence differences by education were the largest in HAPIEE-LT at 20.76% in higher vs 34.91% in less educated adults.

### PRs of depressive symptoms

**Figure 1:**
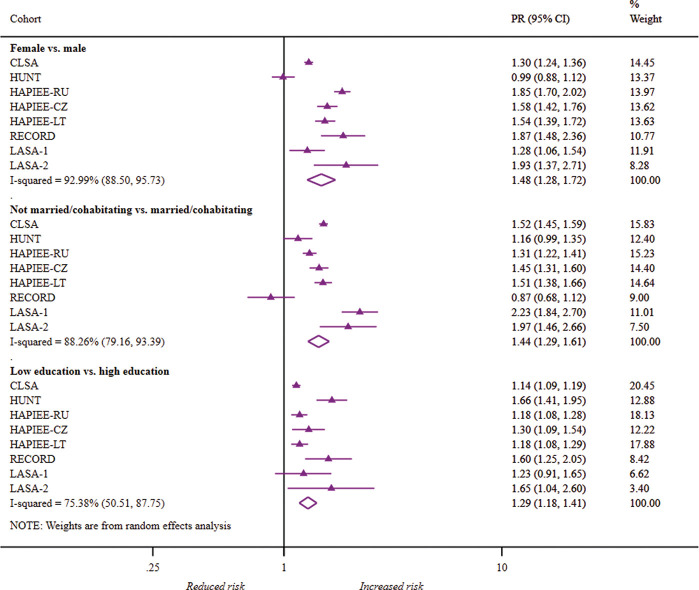
Forest plot of study-specific and pooled prevalence ratios (95% CIs) for probable depression by gender, marital status and education. CLSA, Canadian Longitudinal Study on Aging; CZ, The Czech Republic; HAPIEE, the Health, Alcohol and Psychosocial factors In Eastern Europe; HUNT, the Nord-Trøndelag Health Study; LASA-1, Longitudinal Aging Study Amsterdam—first cohort; LASA-2, Longitudinal Aging Study Amsterdam—second cohort; LT, Lithuania; PR, prevalence ratio; RECORD, the Residential Environment and CORonary heart Disease study, RU, Russia.

**Figure 2:**
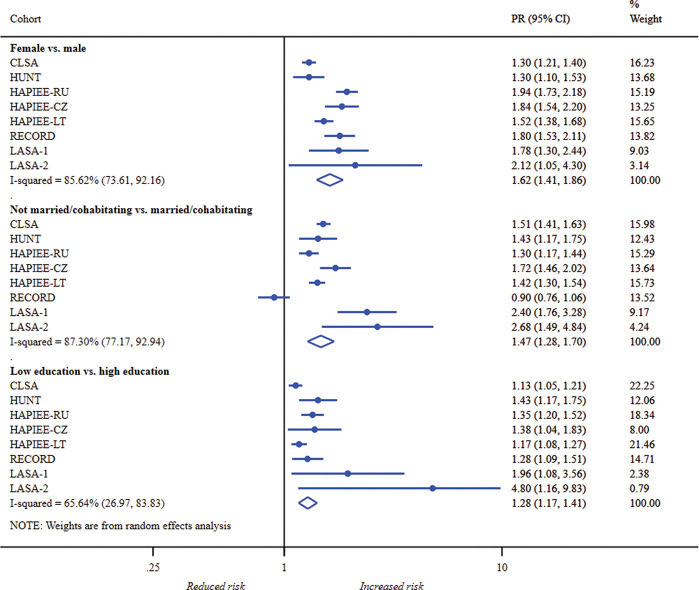
Forest plot of study-specific and pooled prevalence ratios (95% CIs) for depressed affect by gender, marital status and education. CLSA, Canadian Longitudinal Study on Aging; CZ, The Czech Republic; HAPIEE, the Health, Alcohol and Psychosocial factors In Eastern Europe; HUNT, the Nord-Trøndelag Health Study; LASA-1, Longitudinal Aging Study Amsterdam—first cohort; LASA-2, Longitudinal Aging Study Amsterdam—second cohort; LT, Lithuania; PR, prevalence ratio; RECORD, the Residential Environment and CORonary heart Disease study, RU, Russia.

Cohort-specific and pooled PRs in probable depression (figure 1), depressed affect (figure 2) and depressive symptom severity (online supplemental figures S2 and S3) are displayed in forest plots. After mutually adjusting for gender, marital status and education plus age and self-rated health, the PRs of depressive symptoms remained significantly higher in women, unmarried/non-cohabitating adults and those with low education.

Overall, women were 1.48 times more likely to have probable depression than men. The pooled PRs among women were marginally higher for affect and slightly lower for high symptom severity. Cohort-specific PRs were comparable across all settings except in HUNT for probable depression and symptom severity.

Across cohorts, unmarried/non-cohabitating adults were 1.44 and 1.47 times more likely to have probable depression and depressed affect than married counterparts, respectively. The PR for high symptom severity among unmarried/non-cohabitating adults was somewhat lower at 1.33. However, marital status was weakly associated with depression risk in RECORD and HUNT, compared to other cohorts.

The combined PRs by low education ranged from 1.16 for high symptom severity to 1.29 for probable depression. Compared to cohort-specific PRs by gender and marital status, cohort-specific PRs by education were greater than 1 in all eight studies for the three outcomes.

Depressive symptom associations with education were most consistent between cohorts. Although the I^2^ percentage of 75.38 represented considerable cohort variation for probable depression, there was weak evidence of substantial heterogeneity for depressed affect (65.64, 95% CI 26.97 to 83.83), as well as for moderate (23.58, 95% CI 0.00 to 65.02) and high (53.92, 95% CI; 0.00, 79.22) symptom severity. I^2^ and 95% CI percentages were well above 60% for gender and marital status, indicating extensive heterogeneity in these associations between cohorts. For probable depression, heterogeneity for gender and marital status was 92.99 (95% CI 88.50 to 95.73) and 88.26 (95% CI 79.16 to 93.39), respectively.

### Effect modification by population density

Table 3 reports the main effects of population density and its interactions with each determinant on probable depression and depressed affect in the subsample of six cohorts. Higher levels of population density were strongly associated with depression in CLSA. PRs of probable depression and depressed affect increased by 4% and 5% per 1000 increase in residents per square kilometre, respectively.

**Table 3:** Study-specific main effects of population density and interactions with gender, marital status and education on the risk of probable depression and depressed affect

Country and cohort		PR (95% CI) per 1000 residents per square kilometre*	Interaction between gender and population density†	Interaction between marital status and population density†	Interaction between education and population density†
**PRs for probable depression (95% CI)**					
CA	CLSA	1.04 (1.02 to 1.05)	0.99 (0.96 to 1.02) p=0.36	1.01 (0.98 to 1.04) p=0.55	1.02 (0.99 to 1.05) p=0.15
NO	HUNT‡	0.76 (0.68 to 0.85)	0.97 (0.78 to 1.20) p=0.76	1.05 (0.81 to 1.35) p=0.73	0.95 (0.76 to 1.19) p=0.68
CZ	HAPIEE	0.93 (0.82 to 1.06)	0.99 (0.73 to 1.34) p=0.96	0.88 (0.66 to 1.17) *p*=0.39	1.20 (0.78 to 1.84) *p*=0.40
FR	RECORD	1.00 (0.99 to 1.01)	0.9 (0.97 to 1.02) p=0.63	1.00 (0.98 to 1.02) p=0.82	0.99 (0.97 to 1.02) p=0.55
NL	LASA-1	1.00 (0.97 to 1.03)	0.98 (0.91 to 1.05) p=0.51	1.04 (0.98 to 1.12) p=0.20	1.05 (0.93 to 1.19) p=0.44
NL	LASA-2	1.02 (0.99 to 1.05)	0.94 (0.90 to 0.98) p<0.01	0.96 (0.92 to 1.00) p=0.07	0.98 (0.92 to 1.05) p=0.57
**PRs for depressed affect (95% CI)**					
CA	CLSA	1.05 (1.03 to 1.08)	0.98 (0.93 to 1.02) p=0.34	0.99 (0.95 to 1.04) p=0.76	1.02 (0.97 to 1.07) p=0.42
NO	HUNT‡	0.96 (0.88 to 1.04)	1.05 (0.88 to 1.24) p=0.59	0.95 (0.78 to 1.17) p=0.66	1.08 (0.91 to 1.27) p=0.40
CZ	HAPIEE	0.95 (0.85 to 1.06)	1.12 (0.86 to 1.46) *p*=0.41	0.89 (0.69 to 1.16) *p*=0.40	1.08 (0.75 to 1.57) p=0.66
FR	RECORD	1.00 (0.99 to 1.01)	1.00 (0.99 to 1.02) p=0.64	1.00 (0.98 to 1.01) p=0.71	1.00 (0.99 to 1.01) p=0.98
NL	LASA-1	1.00 (0.94 to 1.06)	0.97 (0.86 to 1.11) p=0.69	1.04 (0.93 to 1.17) p=0.48	1.01 (0.84 to 1.21) p=0.94
NL	LASA-2	0.95 (0.87 to 1.03)	1.01 (0.84 to 1.22) p=0.93	0.99 (0.82 to 1.20) p=0.94	0.90 (0.76 to 1.06) p=0.20

*The main effect of population density (per 1000 residents per square kilometre) was adjusted for all three determinants plus age, age squared and self-rated health.

†Interaction effects between population density and each exposure were adjusted for the other two exposures plus age, age squared and self-rated health.

‡The HUNT analyses were based on 10 residents per square kilometre because the maximum population density value was 32 residents per square kilometre in the analytic sample.

CA, Canada; CLSA, Canadian Longitudinal Study on Aging; CZ, The Czech Republic; FR, France; HAPIEE, the Health, Alcohol and Psychosocial factors In Eastern Europe; HUNT, the Nord-Trøndelag Health Study; LASA-1, Longitudinal Aging Study Amsterdam—first cohort; LASA-2, Longitudinal Aging Study Amsterdam—second cohort; LT, Lithuania; NL, The Netherlands; NO, Norway; PR, prevalence ratio; RECORD, the Residential Environment and CORonary heart Disease study, RU, Russia.

Higher population density modified gender and marital status inequalities in LASA-2 but not in the hypothesised direction. PRs for probable depression among women and unmarried persons decreased by 6% (PR: 0.94, 95% CI 0.90 to 0.98) and 4% (PR: 0.96, 95% CI 0.92 to 1.00) for every 1000 increase in residents per square kilometre. After stratifying by population density tertiles in LASA-2, PRs were highest among older women and unmarried persons living in low-density areas, compared to higher-density areas (online supplemental figure S4). Population density did not modify inequalities in other cohorts, including the elder cohort from the same Dutch region (LASA-1). Main and interaction effects for high symptom severity were similarly weak across the six cohorts (results available upon request).

## DISCUSSION

This comparative study of eight European and Canadian cohorts found broadly consistent gender, marital and educational inequalities in mid- to late-life depressive symptoms. Despite our hypothesis, there was weak evidence that population density moderated these inequalities.

### Strengths and limitations

Heterogeneity in measurement of depressive symptoms across cohorts is a major concern. Depressive symptoms were retrospectively harmonised to create three indicators across cohorts using four depression scales (CES-D 20, CES-D 10, HADS-D and QD2A). Although each measured psychological, physical and social symptoms that characterise MDD,^30^ overlap between individual symptoms was moderate. Where same symptoms were collected, scale differences in wording may result in under- or over-reporting of symptoms.^31^ However, some experts have concluded that different depression scales measure depression risk in clinically meaningful ways, provided that they reliably assess the multiple dimensions that characterise depression.^31^ CES-D 10 has demonstrated very good prognostic accuracy in comparison to CES-D 20^20^ which in turn has shown to perform similarly as HADS-D.^32^ Although QD2A has not been examined against HADS-D or the CES-D scales, QD2A has shown a strong-to-moderate correlation with the Zung Self-Rating Depression Scale,^24^ which portrays a highly similar factor structure as the CES-D 20.^33^ Yet, these comparisons were made on specific samples, so the performance of these scales across MINDMAP cohorts is unknown. We addressed this uncertainty by testing associations using several indicators.

Probable depression is most clinically meaningful, but scale-specific thresholds for symptom scores, with different finite ranges, may have identified people with varying pathology or severity. Depressed affect has obvious face validity between studies, but it remains unclear what feeling sad, depressed or downhearted signifies. Comparing associations with a single symptom circumvents the issue that different symptoms may have different risk factors,^34^ and vary in their genetic background.^35^ Indeed, a European-wide study of older adults found that women reported a higher number of affective, but not motivational, symptoms than men.^8^ Nonetheless, depression is considered a latent construct, and its presence is inferred from multiple symptom domains.^31^ Given similar gender, marital and educational inequalities across outcomes, these contentious issues may be waived aside for the present study.

Since population density data at the area level were unavailable for HAPIEE-LT and HAPIEE-RU, we could not explore the moderating hypothesis across the full range of urban settings captured by MINDMAP. Population density appears to have limited capacity to capture important aspects of the urban environment, which may be important for depression. Indeed, health researchers have urged the development of urbanicity scales drawing on a range of reliable measures.^13^ Unfortunately, more nuanced urbanicity measures were unavailable across cohorts at this stage of the project. Furthermore, cross-cohort findings may be influenced by cohort and period effects since participants were studied from 1992–1993 (LASA-1) to 2010–2015 (CLSA). Despite this time span, the magnitude of cross-cohort inequalities aligns with several European reports which used concurrent data between countries,^6–8^ suggesting that depression inequalities may be robust to broad secular changes.

### Consistency with existing evidence

The magnitude of depression inequalities found across MINDMAP studies is remarkably consistent with high-quality evidence from several reviews.^3–5^ A meta-analysis of five prospective studies reported that community-dwelling women aged ≥50 years have a 1.4 higher odds of depression than men, but stated weaker pooled ORs of 1.0 (95% CI 0.8 to 1.3) and 1.5 (95% CI 0.8 to 2.8) by marital status and education, respectively.^3^ Larger systematic reviews and meta-analyses of cross-sectional and prospective studies on adults aged ≥55 years, however, found increased risks associated with never married (relative risk (RR) 1.32) and widowed (RR 1.49) statuses^4^ as well as with low education (OR 1.58) on prevalent depression.^5^ Although we analysed self-reported symptoms in the absence of clinically diagnosed cases, considered to be more valid measures of depressive disorders,^36^ our findings demonstrate that the MINDMAP-harmonised data set is a solid resource for comparative ageing and mental well-being research.^14^

In a study of adults aged ≥50 years from Northern, Western and Southern Europe, PRs for probable depression, measured by the European Depression (EURO-D) scale, risks among females were lowest in Denmark (1.91) and higher in Spain (3.89). PRs comparing low to high educational groups varied widely from 1.70 (Greece) to 3.02 (France).^7^ Among adults aged ≥65 years from similar parts of Europe, never married, widowed and divorced/separated adults reported a higher number of depressive symptoms than married adults, but only in 9 of 13 sites.^8^ Few European studies on mid- to late-life depression included Central and Eastern Europe (CEE), but larger gender^6^ and educational^37^ inequalities have been reported in CEE than in Northern countries including Norway, among adults aged 18–75^6^ and 60–80 years,^37^ respectively. Altogether, these comparative findings^6–8 37^ align with the cross-cohort variation of depression inequalities reported in our study. Since these studies measured depressive symptoms using a single scale across countries,^6–8 37^ this provides further support that cross-cohort variation in MINDMAP is not driven by the abovementioned measurement issues.

Our study found that population density was weakly associated with depression, and consequently did not modify the strong effects of gender, marital status and education on depression. Although the higher depression risk among older adults in urban versus rural areas is well established,^2^ there is limited and mixed evidence of a graded increase in depression by increasing population density.^38 39^ Probable depression risk among women and unmarried/non-cohabitating adults decreased in higher-density areas in LASA-2. This could indicate greater convergence within private or social life between men and women^6^ and greater social integration of older single persons^8^ with higher levels of urbanicity. Since these results were not replicated in other cohorts, including LASA-1, which consisted of adults born decades earlier from the same regions, the LASA-2 results may be due to confounding or selection biases. Despite some ecological correlation between population density and depression inequalities, both of which were lower in HUNT than in other cohorts, inequalities within cohorts were unmodified by densification, suggesting that people’s position in society are ‘fundamental causes’ of inequalities irrespective of the social context.^40^

## CONCLUSION

Given strong mid- to late-life depression inequalities amidst increasing rates of urbanicity in Europe and Canada, future work should assess the interplay between putative risk factors and specific urban municipal-level determinants and living conditions,^9^ which may influence older adults’ mental health.

**What is already known on this subject**

- Gender, marital and educational inequalities in mid- to late-life depression are well established, but the magnitude of these inequalities may vary between European and North American countries due to differences in urbanicity.
- The share of older European and North American adults residing in cities has grown exponentially over the last several decades. Living in more densely populated cities may exacerbate depression risks associated with female gender, unmarried/divorced/widowed status and low educational attainment.
- This study hypothesised that increasing levels of population density, a marker of urbanicity, would magnify mid- to late-life depression inequalities.

**What this study adds**

- This comparative study of European and Canadian ageing cohorts found strong gender, marital and educational inequalities in depressive symptoms, and the magnitude varied between cohorts.
- However, population density, measured by the number of residents per square kilometre, was weakly associated with depression risk across cohorts, and thus did not modify depression inequalities.
