# Supplementary material for: Gender, marital and educational inequalities in mid- to late-life depressive symptoms: cross-cohort variation and moderation by urbanicity degree
Source: J Epidemiol Community Health. 2020 Nov 4;75(5):442–9. doi: 10.1136/jech-2020-214241 (PMC8053350; doi:10.1136/jech-2020-214241)
Supplement: Supplementary data [file jech-2020-214241supp001.pdf]

Title: Gender, marital and educational inequalities in mid-late life depressive symptoms: Cross-cohort variation and moderation by urbanicity degree

Author names: Milagros A. Ruiz, Mariëlle A. Beenackers, Dany Doiron, Asli Gurer, Aliou Sarr, Nazmul Sohel, Erik J. Timmermans, Rita Wissa, Basile Chaix, Martijn Huisman, Steinar Krokstad, Ruzena Kubinova, Sofia Malyutina, Parminder Raina, Abdonas Tamosiunas, Frank J. van Lenthe, Martin Bobak

Correspondence to: Dr Milagros Ruiz, Research Department of Epidemiology and Public Health, University College London, 1-19 Torrington Place, London WC1E 7HB, UK, m.a.ruiz@ucl.ac.uk, +44 (0)20 7679 8252

Supplementary Table S1 – Harmonization of depressive symptoms 2

Supplementary Figure S1 – Selection diagram of the analytic samples 3

Supplementary Table S2 – Age-standardised study-specific prevalence of depressive symptom severity by gender, marital status and education 4

Supplementary Figure S2 – Forest plot of study-specific and pooled prevalence ratios (95% CIs) for moderate depressive symptom severity by gender, marital status and education 5

Supplementary Figure S3 – Forest plot of study-specific and pooled prevalence ratios (95% CIs) for high depressive symptoms severity by gender, marital status and education 6

Supplementary Figure S4 – Prevalence ratios (95% CIs) for probable depression by gender (left) and marital status (right) stratified by population density tertiles in LASA-2 7

Supplementary Table S1 – Harmonization of depressive symptoms

<table>
<tr><th rowspan="2"></th><th colspan="8">Country[*] and cohort[†]</th></tr>
<tr><th>CA
CLSA</th><th>NO
HUNT</th><th>RU
HAPIEE</th><th>CZ
HAPIEE</th><th>LT
HAPIEE</th><th>FR
RECORD</th><th>NL
LASA-1</th><th>NL
LASA-2</th></tr>
<tr><td>Depressive symptom severity</td><td>The CES-D 10[1] depression scores (0-30) were grouped into study-specific tertiles defined as low (0-2), medium (3-6), and high (7-30). Scores were calculated for participants with data on at least 8 items (out of a possible 10). If 1 or 2 items were missing, scores on the completed items were summed and divided by the number of items completed, then multiplied by 10. If participants missed 3 or more items, scores were not calculated and coded as missing.</td><td>The HADS-D[2] depression scores (0-21) were grouped into study-specific tertiles defined as low (0-1), medium (2-4), and high (5-21). Scores were used as provided by the HUNT study.</td><td colspan="2">The CES-D 20[3] depression scores (0-60) were grouped into population-specific tertiles for each cohort:
HAPIEE-RU
• Low (0-8)
• Medium (9-12)
• High (13-60)
HAPIEE-CZ
• Low (0-5)
• Medium (6-11)
• High (12-60)
Scores were calculated for participants with data on least 16 items (out of a possible 20). If 1 or more items were missing, scores on the completed items were summed and divided by the number of items completed, then multiplied by 20. If participants missed 5 or more items, scores were not calculated and coded as missing.</td><td>The CES-D 10[4] depression scores (0-10) were grouped into population-specific tertiles for the Lithuanian HAPIEE cohort: low (0-1), medium (2-3), and high (4-10). Scores were calculated for participants with data on least 8 items (out of a possible 10). If 1 or 2 items were missing, scores on the completed items were summed and divided by the number of items completed, then multiplied by 10. If participants missed 3 or more items, scores were not calculated and coded as missing.</td><td>The QD2A[5] depression scores (0-13) in RECORD were grouped into study-specific tertiles defined as low (0), medium (1-2), and high (3-13). Scores were calculated for participants with data on at least 11 items (out of a possible 13). If 1 or 2 items were missing, scores on the completed items were summed and divided by the number of items completed and multiplied by 13. If participants missed 3 or more items, scores were not calculated and coded as missing.</td><td colspan="2">The CES-D 20[3] depression scores (0-60) in LASA were grouped into study-specific tertiles defined as low (0-3), medium (4-9), and high (10-60). Scores were calculated for participants with data on at least 16 items (out of a possible 20). If 1 or more items were missing, scores on the completed items were summed and divided by the number of items completed, then multiplied by 20. If participants missed 5 or more items, scores were not calculated and coded as missing.</td></tr>
<tr><td>Probable depression</td><td>Participants with CES-D 10 scores of 10 or higher (out of a possible 30) were defined as cases as per the scale-specific threshold.[1]</td><td>Participants with HADS-D depression scores of 11 or higher (out of a possible 21) were defined as cases as per the scale-specific threshold.[2 6]</td><td colspan="2">Participants with CESD-20 scores of 16 or higher (out of a possible 60) were defined as cases as per the scale-specific threshold.[7]</td><td>Participants with CES-10 scores of 4 or higher (out of a possible 10) were defined as cases as per the scale-specific threshold.[4]</td><td>Participants with QD2A depression scores of 7 or higher (out of a possible 13) were defined as cases as per the scale-specific threshold.[5]</td><td colspan="2">Participants with CESD-20 scores of 16 or higher (out of a possible 60) were defined as cases as per the scale-specific threshold.[7]</td></tr>
<tr><td>Depressed affect</td><td>Based on a 4-point ('rarely or never', 'some of the time', 'occasionally', 'all of the time') item response to "I felt depressed" from the CES-D scale, 10-item version. Depressed affect was identified for those who responded 'occasionally' or 'all of the time'.</td><td>Based on a 7-point ('very downhearted', 'downhearted', 'somewhat downhearted', 'some of both', 'somewhat cheerful', 'cheerful', 'very cheerful') item response to "Would you say you are usually cheerful or downhearted?" from the ADI-4[8] anxiety and depression scale. Depressed affect was identified for those who responded from 'very downhearted' to 'somewhat downhearted'.</td><td colspan="2">Based on a 4-point ('less than 1 day', '1-2 days', '3-4 days', '5-7 days') item response to "I felt sad" from the CES-D scale, 20-item version. Depressed affect was identified for those who responded '3-4 days' or '5-7 days'.</td><td>Based on a yes/no item response to "I felt sad" from the CES-D scale, 10-item version.</td><td>Based on a yes/no item response to "I feel sad at present" from the QD2A depression scale.</td><td colspan="2">Based on a 4-point ('rarely or none of the time', 'some or a little of the time', 'occasionally or a moderate amount of time', 'most or all of the time') item response to "I felt sad" from the CES-D scale, 20-item version. Depressed affect was identified for those who responded 'occasionally or a moderate amount of time' or 'most or all of the time'.</td></tr>
</table>

Supplementary Figure S1 – Selection diagram of the analytic samples

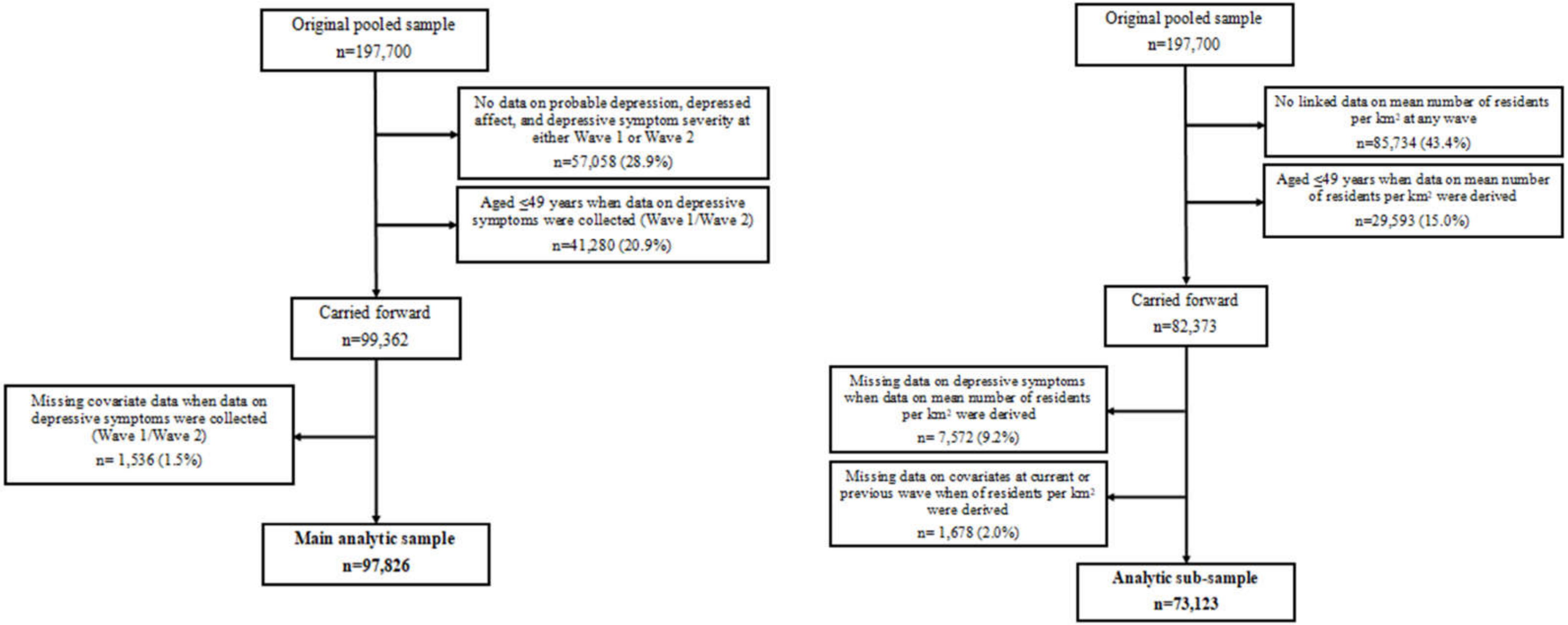

Supplementary Table S2 – Age-standardised study-specific prevalence of depressive symptom severity by gender, marital status and education

Country* and cohort†		Male	Female	Married/ cohabitating	Not married/ cohabitating	High education	Low education
**Low symptom severity**							
CA	CLSA	34.74 [33.70, 35.78]	27.92 [26.98, 28.87]	33.55 [32.65, 34.45]	24.84 [23.71, 25.98]	32.50 [31.63, 33.37]	28.17 [26.91, 29.43]
NO	HUNT	22.07 (21.24, 22.89)	24.09 (23.32, 24.86)	22.74 (22.12, 23.37)	24.69 (23.22, 26.15)	24.38 (23.20, 25.56)	22.69 (22.04, 23.35)
RU	HAPIEE	49.32 (46.83, 51.80)	34.51 (32.10, 36.91)	45.19 (43.05, 47.33)	31.72 (28.82, 34.62)	44.74 (41.19, 48.28)	40.07 (38.08, 42.05)
CZ	HAPIEE	34.93 (32.73, 37.12)	27.77 (25.51, 30.02)	34.02 (32.10, 35.93)	24.39 (21.65, 27.13)	39.98 (35.64, 44.31)	30.12 (28.43, 31.81)
LT	HAPIEE	50.82 (49.14, 52.49)	41.35 (39.66, 43.05)	54.69 (53.15, 56.22)	31.23 (29.24, 33.23)	51.40 (49.67, 53.13)	40.48 (38.76, 42.2)
FR	RECORD	53.55 (51.17, 55.93)	38.12 (35.40, 40.84)	48.20 (46.03, 50.37)	46.05 (42.95, 49.15)	49.13 (46.36, 51.89)	46.41 (44.06, 48.77)
NL	LASA-1	53.44 (51.16, 55.72)	24.63 (22.58, 26.68)	43.21 (30.27, 56.15)	23.85 (20.68, 27.03)	49.99 (45.76, 54.21)	29.42 (27.76, 31.07)
NL	LASA-2	49.60 (30.22, 68.98)	14.03 (12.09, 15.97)	29.64 (17.48, 41.80)	21.52 (15.87, 27.18)	44.08 (18.82, 69.34)	25.25 (10.21, 40.30)
**Moderate symptom severity**							
CA	CLSA	37.85 [36.77, 38.93]	36.77 [35.73, 37.81]	38.00 [37.04, 38.96]	34.94 [33.67, 36.21]	37.84 [36.93, 38.75]	36.07 [34.70, 37.43]
NO	HUNT	36.93 (35.96, 37.90)	37.33 (36.44, 38.23)	37.57 (36.81, 38.34)	34.43 (32.83, 36.04)	39.84 (38.45, 41.24)	36.46 (35.70, 37.22)
RU	HAPIEE	22.77 (20.54, 25.01)	18.32 (16.47, 20.17)	21.05 (19.27, 22.82)	19.31 (16.73, 21.89)	22.62 (19.51, 25.73)	19.69 (18.04, 21.33)
CZ	HAPIEE	33.67 (31.52, 35.83)	29.58 (27.21, 31.95)	32.66 (30.78, 34.55)	28.18 (25.28, 31.08)	34.31 (30.16, 38.47)	31.10 (29.39, 32.8)
LT	HAPIEE	23.78 (22.28, 25.27)	28.89 (27.32, 30.45)	26.93 (25.57, 28.29)	26.69 (24.79, 28.59)	27.84 (26.29, 29.39)	24.61 (23.08, 26.15)
FR	RECORD	30.70 (28.45, 32.96)	29.91 (27.37, 32.45)	30.15 (28.13, 32.18)	29.89 (26.88, 32.89)	31.01 (28.38, 33.64)	29.41 (27.22, 31.61)
NL	LASA-1	26.91 (24.70, 29.11)	46.28 (44.17, 48.39)	37.13 (24.19, 50.06)	31.82 (28.41, 35.22)	29.66 (25.15, 34.17)	45.51 (43.90, 47.13)
NL	LASA-2	35.47 (16.10, 54.84)	52.68 (33.15, 72.21)	51.31 (39.15, 63.46)	29.83 (23.52, 36.13)	38.26 (13.03, 63.48)	49.22 (34.17, 64.27)
**High symptom severity**							
CA	CLSA	27.41 [26.42, 28.41]	35.30 [34.27, 36.33]	28.45 [27.55, 29.36]	40.22 [38.91, 41.52]	29.66 [28.80, 30.52]	35.76 [34.40, 37.12]
NO	HUNT	41.00 (40.01, 41.99)	38.57 (37.68, 39.47)	39.68 (38.91, 40.46)	40.88 (39.22, 42.54)	35.78 (34.40, 37.15)	40.85 (40.07, 41.62)
RU	HAPIEE	27.91 (25.43, 30.39)	47.17 (44.63, 49.71)	33.77 (31.62, 35.91)	48.97 (45.73, 52.21)	32.64 (29.26, 36.03)	40.25 (38.15, 42.34)
CZ	HAPIEE	31.40 (29.27, 33.53)	42.65 (40.06, 45.24)	33.32 (31.45, 35.19)	47.43 (44.17, 50.68)	25.71 (21.80, 29.62)	38.78 (36.98, 40.59)
LT	HAPIEE	25.40 (24.18, 26.63)	29.76 (28.19, 31.33)	18.39 (17.19, 19.58)	42.07 (40.10, 44.04)	20.76 (19.36, 22.16)	34.91 (33.39, 36.42)
FR	RECORD	15.75 (14.04, 17.45)	31.97 (29.33, 34.62)	21.65 (19.82, 23.47)	24.06 (21.23, 26.90)	19.86 (17.60, 22.12)	24.17 (22.10, 26.24)
NL	LASA-1	19.66 (17.61, 21.71)	29.09 (26.98, 31.19)	19.66 (17.66, 21.66)	44.33 (40.68, 47.98)	20.35 (16.23, 24.48)	25.07 (23.48, 26.66)
NL	LASA-2	14.93 (12.47, 17.39)	33.29 (13.75, 52.83)	19.05 (9.10, 29.00)	48.65 (41.75, 55.56)	17.66 (13.82, 21.50)	25.53 (10.49, 40.58)

*Country abbreviations represent Canada (CA), Norway (NO), Russia (RU), the Czech Republic (CZ), Lithuania (LT), France (FR) and the Netherlands (NL).

†Cohort acronyms stand for the Canadian Longitudinal Study on Aging (CLSA), the Nord-Trøndelag Health Study (HUNT), the Health, Alcohol and Psychosocial factors in Eastern Europe (HAPIEE) study, the Residential Environment and CORonary heart Disease (RECORD) study, the Longitudinal Aging Study Amsterdam – 1st cohort (LASA-1) and the Longitudinal Aging Study Amsterdam – 2nd cohort (LASA-2).

Supplementary Figure S2 – Forest plot of study-specific and pooled prevalence ratios (95% CIs) for moderate depressive symptom severity by gender, marital status and education

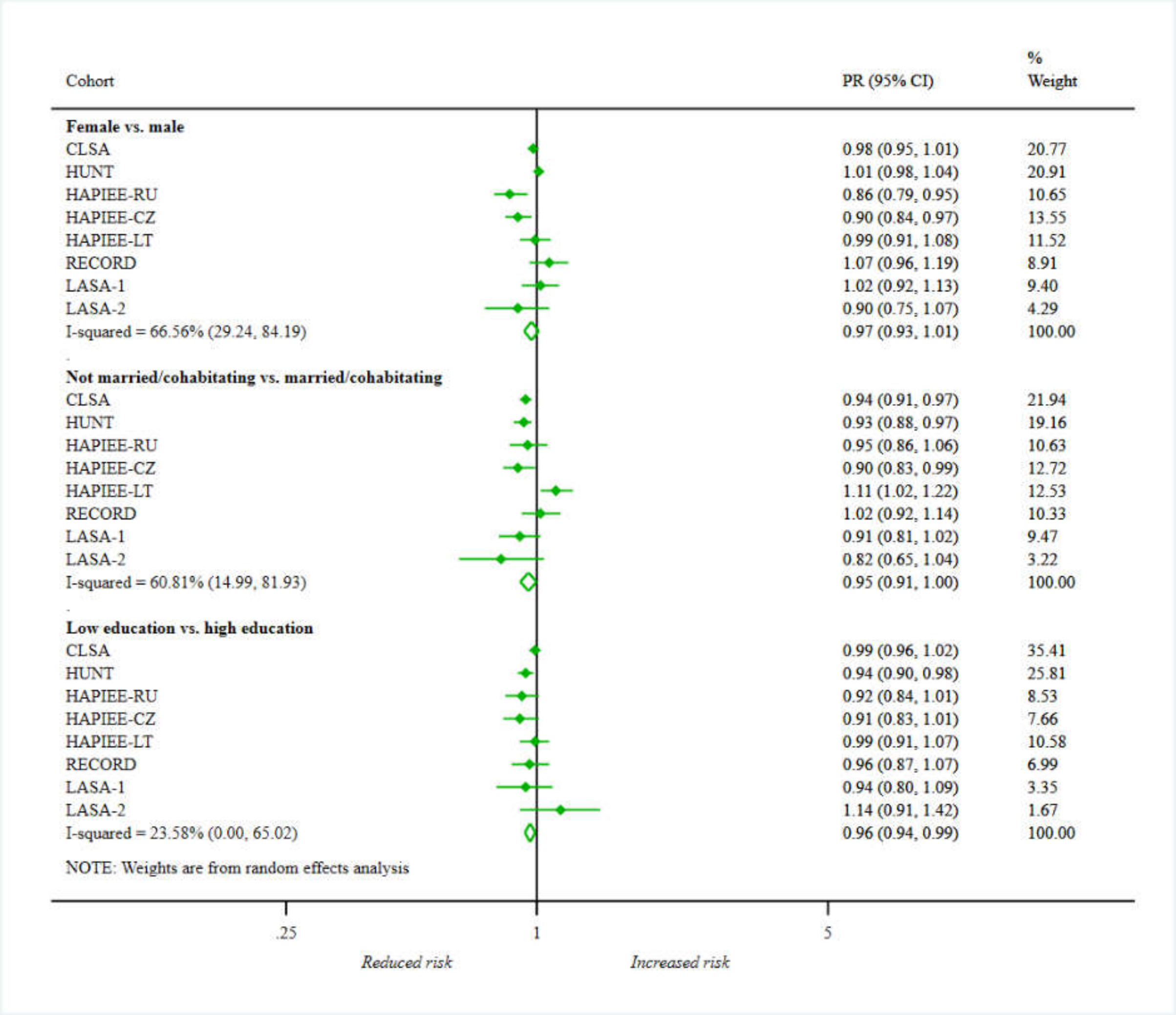

Supplementary Figure S3 – Forest plot of study-specific and pooled prevalence ratios (95% CIs) for high depressive symptoms severity by gender, marital status and education

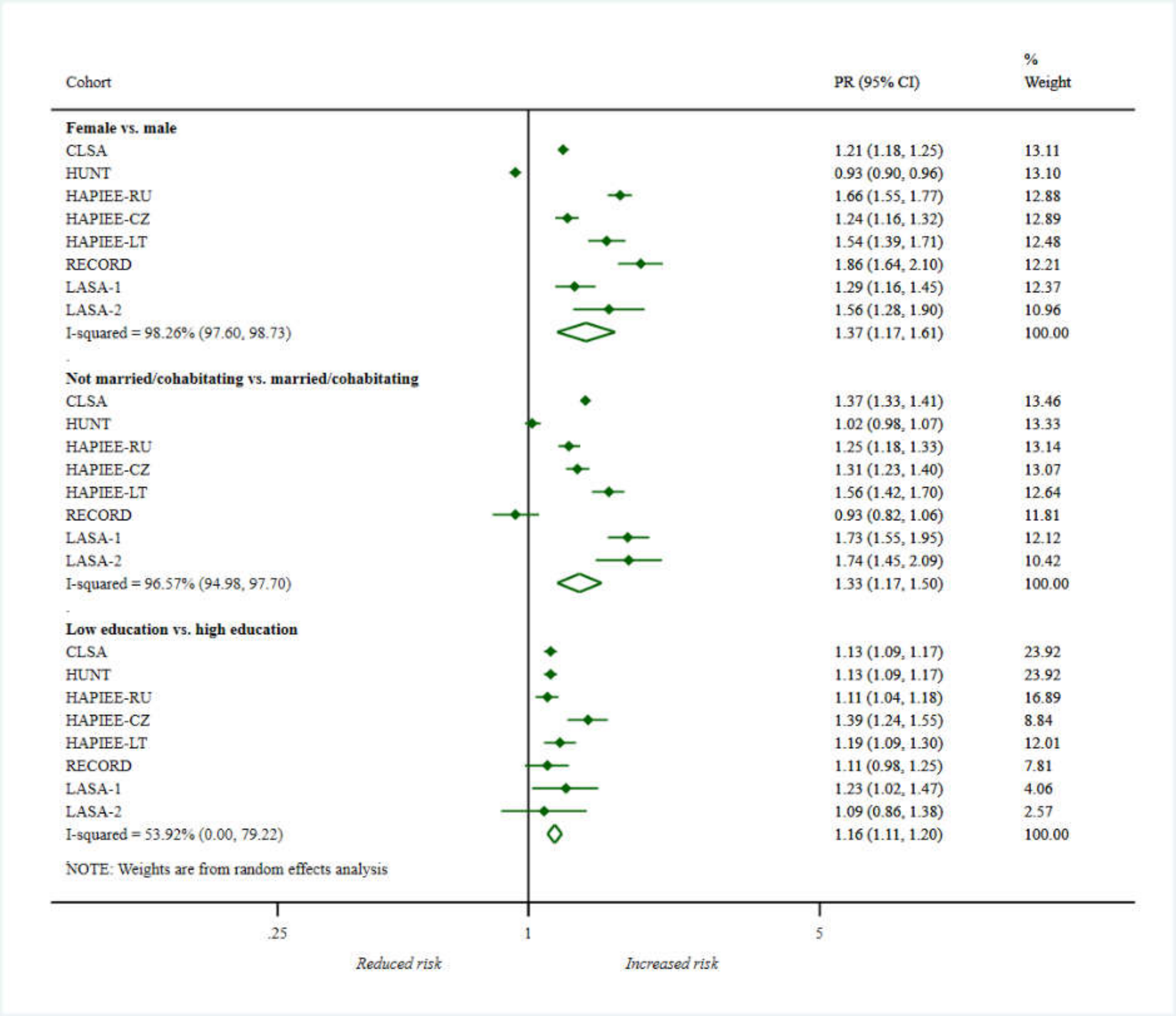

Supplementary Figure S4 – Prevalence ratios (95% CIs) for probable depression by gender (left) and marital status (right) stratified by population density tertiles in LASA-2

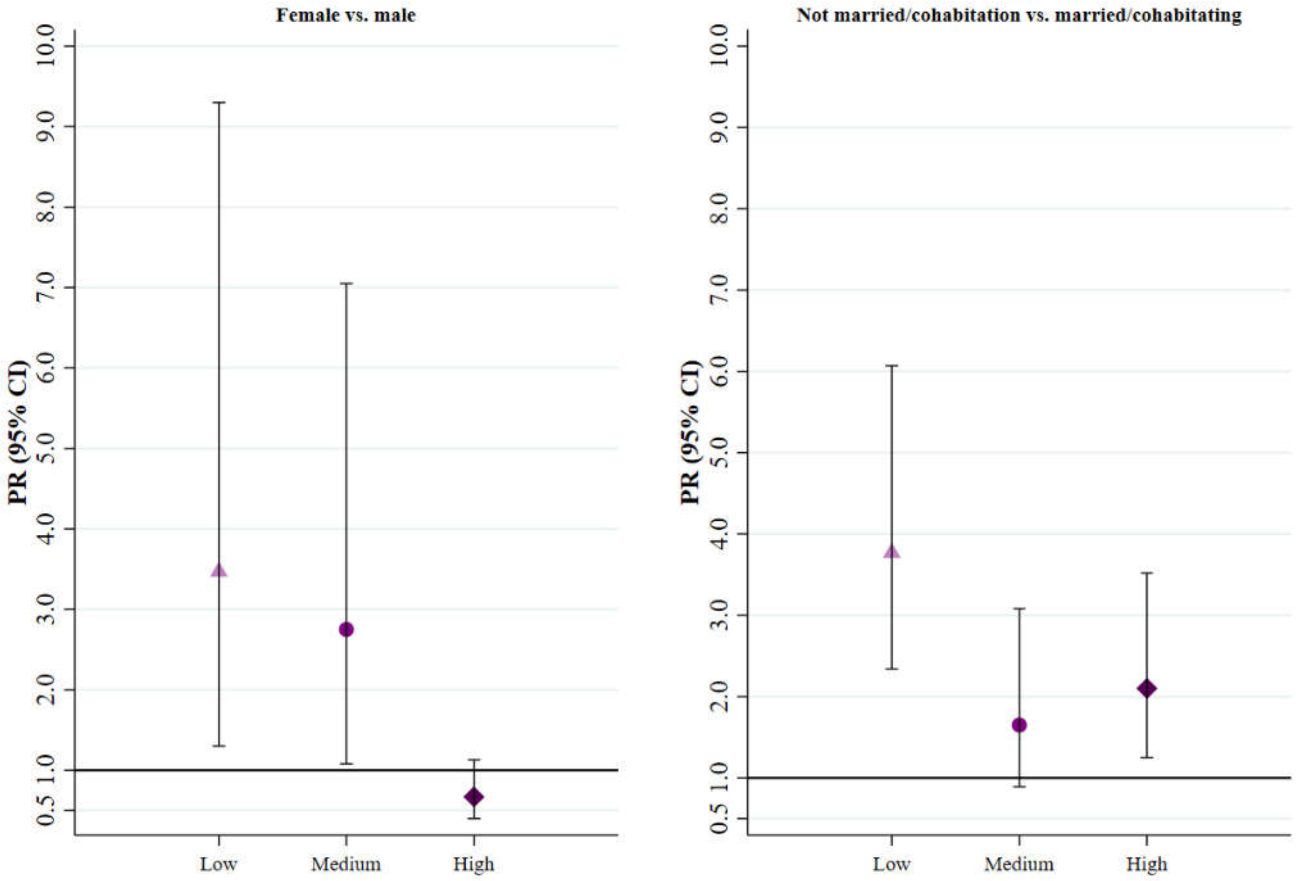

The population density cut-off points are 9-3,241 (low), 3,246-5,339 (medium) and 5,355-26,763 (high) residents per km² in LASA-2.

References

1. Andresen EM, Malmgren JA, Carter WB, et al. Screening for depression in well older adults: evaluation of a short form of the CES-D (Center for Epidemiologic Studies Depression Scale). *Am J Prev Med* 1994;10(2):77-84.
2. Zigmond AS, Snaith RP. The hospital anxiety and depression scale. *Acta Psychiatr Scand* 1983;67(6):361-70.
3. Radloff LS. The Center for Epidemiologic Studies Depression Scale: a self-report depression scale for research in the general population *Appl Psychol Meas* 1977;1(3):385-401. doi: 10.1177/014662167700100306
4. Irwin M, Artin KH, Oxman MN. Screening for depression in the older adult: criterion validity of the 10-item Center for Epidemiological Studies Depression Scale (CES-D). *Arch Intern Med* 1999;159(15):1701-4.
5. Pichot P. A Self-Report Inventory on Depressive Symptomatology (QD2) and Its Abridged Form (QD2A). In: Sartorius N, Ban TA, eds. Assessment of Depression. Berlin, Heidelberg: Springer Berlin Heidelberg 1986:108-22.
6. Snaith RP. The Hospital Anxiety And Depression Scale. *Health Qual Life Outcomes* 2003;1:29. doi: 10.1186/1477-7525-1-29
7. Weissman MM, Sholomskas D, Pottenger M, et al. Assessing depressive symptoms in five psychiatric populations: a validation study. *Am J Epidemiol* 1977;106(3):203-14. doi: 10.1093/oxfordjournals.aje.a112455
8. Gustad LT, Laugsand LE, Janszky I, et al. Symptoms of anxiety and depression and risk of acute myocardial infarction: the HUNT 2 study. *Eur Heart J* 2014;35(21):1394-403. doi: 10.1093/eurheartj/eht387
